# Perioperative Systemic Therapy in Localized Renal Cell Carcinoma: Current Evidence and Future Directions

**DOI:** 10.7759/cureus.99211

**Published:** 2025-12-14

**Authors:** José Pereira, Mário Pereira-Lourenço, Ana Maria Ferreira, Rita Marques, Ricardo Godinho, João Pedro Peralta, Bruno Jorge Pereira, Paulo Conceicao, Carlos Rabaca

**Affiliations:** 1 Urology, Instituto Português de Oncologia de Coimbra, Coimbra, PRT

**Keywords:** adjuvant therapy, biomarkers, checkpoint inhibitors, inferior vena cava tumor thrombus, minimal residual disease, neoadjuvant therapy, perioperative therapy, renal cell carcinoma, vegfr tyrosine kinase inhibitors

## Abstract

Localized renal cell carcinoma (RCC) carries a substantial risk of recurrence after nephrectomy, particularly in patients with adverse pathological features. Perioperative systemic therapy aims to eradicate micrometastatic disease. This review summarizes current evidence for neoadjuvant, adjuvant, and perioperative strategies in localized RCC, and outlines how emerging biological insights may reshape trial design and clinical practice. We conducted a narrative, non-systematic review of phase II-III studies and conference presentations through August 2025, examining trials that reported disease-free or recurrence-free and overall survival, perioperative feasibility, and translational endpoints, with data cross-checked against full texts and meeting abstracts. Adjuvant pembrolizumab improves disease-free and overall survival in selected high‑risk clear‑cell RCC and is now standard of care, whereas results from vascular endothelial growth factor receptor (VEGFR) tyrosine kinase inhibitors (TKIs) and mTOR inhibitors have been inconsistent across trials, with most studies failing to demonstrate a survival benefit. Neoadjuvant immune checkpoint inhibitor (ICI), TKI, and immuno-oncology-tyrosine kinase inhibitor (IO-TKI) regimens are feasible and generally safe without delaying surgery; radiographic shrinkage is modest, but clinically meaningful surgical facilitation has been observed, particularly in cases with inferior vena cava tumor thrombus. To date, no randomized neoadjuvant trial has improved disease-free or overall survival, and the only completed phase III perioperative trial (PROSPER RCC) did not improve recurrence‑free survival, underscoring the need to better understand the optimal duration, timing, and patient selection for perioperative immunotherapy. Emerging biomarkers, including circulating KIM-1, ctDNA, and CD39^+^ and TCF-1^+^ CD8 states, may enable risk stratification and treatment tailoring but remain exploratory. Overall, adjuvant pembrolizumab is practice-changing for high-risk clear-cell RCC, while neoadjuvant and perioperative approaches remain investigational. Advancing the field will require biomarker‑driven patient selection supported by adaptive or platform trial designs capable of rapidly evaluating multiple strategies.

## Introduction and background

Renal cell carcinoma (RCC) encompasses a heterogeneous group of malignancies with diverse histological subtypes and molecular alterations, contributing to its variable biological behavior and clinical outcomes [1]. Globally, RCC accounts for more than 430,000 new cases annually and represents approximately 2-3% of all cancers, with rising incidence in industrialized countries [2].

Surgical resection remains the cornerstone of treatment for localized RCC. However, a substantial proportion of patients with high-risk features (including pathological stage ≥pT2, nodal involvement, sarcomatoid differentiation, or venous tumor thrombus) will ultimately relapse. Such high-risk features are consistently associated with higher recurrence rates in prospective cohorts and across adjuvant trial populations. Among patients with high-risk features eligible for adjuvant trials, five-year disease-free survival (DFS) typically ranges from 45% to 54%, underscoring the need for more effective perioperative strategies [3,4].

The development of vascular endothelial growth factor receptor (VEGFR)-targeted therapies and immune checkpoint inhibitors (ICIs) has revolutionized the management of metastatic RCC and prompted investigation in earlier disease settings [5]. In the adjuvant context, results have been heterogeneous: KEYNOTE-564 demonstrated significant improvements in DFS and overall survival (OS) with pembrolizumab, whereas CheckMate-914 (nivolumab plus ipilimumab) and IMmotion010 (atezolizumab) failed to meet their primary endpoints [6-8]. These divergent outcomes may reflect differences in eligibility criteria, pathological risk definitions, programmed death-ligand 1 (PD-L1) requirements, and treatment duration across trials, complicating cross-trial comparisons and endpoint interpretation.

Neoadjuvant therapy has gained momentum due to its biological appeal. Administering systemic treatment before nephrectomy may leverage the intact antigenic repertoire of the tumor to enhance T-cell priming and systemic immune activation. Additionally, the neoadjuvant window provides a unique opportunity for translational research, enabling paired pre- and post-treatment sampling to assess pathological responses and identify biomarkers of sensitivity or resistance. Yet, clinical evidence remains limited, and uncertainties regarding optimal design, biomarker integration, and surgical timing have hindered widespread adoption [5,9-12].

This review provides a clinically oriented synthesis of perioperative systemic therapy in localized RCC - who to treat, what to use, when to treat (neoadjuvant, adjuvant, or both), and how to integrate regimens within multidisciplinary care. We summarize efficacy and feasibility data and outline how emerging biomarkers may refine patient selection.

## Review

Methods

This narrative, non-systematic review used purposive evidence selection to summarize phase II-III interventional trials of neoadjuvant, adjuvant, or perioperative systemic therapy in adults with nonmetastatic RCC undergoing curative-intent nephrectomy, together with clinically relevant translational reports. Sources included PubMed/MEDLINE, ClinicalTrials.gov, the Cochrane Library, and meeting proceedings from the American Society of Clinical Oncology (ASCO), the European Society for Medical Oncology (ESMO), and the Society of Urologic Oncology (SUO) from database inception through July 31, 2025. Search terms were combinations of “renal cell carcinoma,” “localized,” “neoadjuvant,” “adjuvant,” “perioperative,” “systemic therapy,” “immune checkpoint inhibitor,” “VEGFR TKI,” “IO-TKI,” “mTOR inhibitor,” and “biomarker.” No language or date restrictions were applied; non-human studies were excluded. Reference lists of included articles and relevant reviews were also screened to identify additional studies.

Eligible designs were randomized controlled trials and prospective single-arm phase II studies. We prioritized completed studies reporting oncologic outcomes (disease-free/recurrence-free survival (RFS) and/or OS), perioperative feasibility (e.g., time to surgery, treatment completion), and, when available, translational endpoints. We excluded studies limited to the metastatic setting, retrospective series without a defined perioperative intervention, and noninterventional biomarker-only reports.

Because of substantial heterogeneity in designs, populations, endpoint definitions (DFS vs. RFS vs. event-free survival (EFS); investigator vs. blinded independent central review), follow-up maturity, and treatment exposure, no meta-analysis or indirect cross-trial comparisons were performed. This review was not designed for the Preferred Reporting Items for Systematic Reviews and Meta-Analyses (PRISMA)-style reproducibility; no protocolized comprehensive search or dual-review screening workflow was undertaken, and screening counts/flow diagrams were not maintained. For phase III randomized trials, we qualitatively considered risk of bias (sequence generation/allocation concealment, blinding, completeness of follow-up, and selective reporting) based on primary publications. To standardize reporting, for randomized phase III trials, we present the prespecified primary-endpoint hazard ratio with 95% confidence interval and P-value, and provide median follow-up when available; when multiple effect measures were reported, primary outcomes took precedence. For prospective single-arm studies, we summarize prespecified efficacy and feasibility endpoints. All hazard ratios, confidence intervals, and P-values were extracted verbatim from peer-reviewed articles or full meeting abstracts.

Biological rationale for perioperative systemic therapy in localized RCC

Therapeutic Objective and Constraints

Perioperative therapy seeks to eradicate occult micrometastases and reduce recurrence after nephrectomy. Two biologic windows can be exploited: (i) the neoadjuvant setting, in which the intact primary tumor provides abundant antigen and tumor-draining lymphatics for T-cell priming, and (ii) the adjuvant setting, when tumor burden is lowest and residual disease may be most vulnerable to immune surveillance. Window-of-opportunity studies and perioperative immunotherapy reviews across cancers support this concept, while also highlighting the need to avoid surgery-related immunosuppression through careful timing and early recognition of immune-related toxicities [5,13].

Why Neoadjuvant Therapy Is Biologically Attractive

Keeping the primary tumor in situ may enhance clonal priming and broaden anti-tumor T-cell responses. Experience from other solid tumors supports this idea. In resectable non-small-cell lung cancer, neoadjuvant nivolumab plus chemotherapy increased pathological complete response (pCR) and prolonged EFS, with an OS benefit. In melanoma, neoadjuvant nivolumab-ipilimumab improved EFS compared with adjuvant-only approaches. Together, these data suggest that engaging the immune system early can improve clinical consequences [14-16].

In RCC, early phase studies point in the same direction. Short-course pre-operative nivolumab was delivered without delaying surgery and was associated with increased intratumoral immune infiltration [17]. Immuno-oncology-tyrosine kinase inhibitor (IO-TKI) combinations such as sitravatinib-nivolumab and avelumab-axitinib produced radiographic downsizing and on-treatment immune activation (increased CD8 infiltration, PD-L1 upregulation) [11,18]. In anatomically complex disease, targeted TKIs and peri-IO-TKI regimens have reduced the level and extent of inferior vena cava (IVC) tumor thrombus, enabling less morbid surgical approaches [10,19]. Together, these data reinforce the biological plausibility and clinical feasibility of neoadjuvant therapy in RCC.

VEGF-driven signaling supports an immunosuppressive tumor microenvironment, whereas VEGF blockade can improve immune infiltration and enhance the activity of PD-1/PD-L1 inhibitors. This provides the rationale for IO-TKI combinations in the perioperative setting [20,21].

What Adjuvant Therapy Adds

Adjuvant therapy targets minimal residual disease (MRD) when the tumor burden is lowest. In RCC, pembrolizumab improved DFS and later OS, providing proof of principle that post-operative PD-1 blockade can alter the natural history of the disease in selected high-risk patients [22]. By contrast, adjuvant atezolizumab and nivolumab-ipilimumab failed to improve DFS, underscoring that efficacy is agent- and biology-dependent and reinforcing the need for biomarker-driven selection [7,8].

Translational Markers and Exploratory Signals for Perioperative Personalization

The perioperative window provides direct access to both tumor tissue and blood, enabling real-time assessment of immune dynamics and exploratory biomarker discovery. The following markers represent investigational signals described in early translational studies, rather than validated predictors of clinical benefit:

CD39^+^ CD8 T cells: CD39 marks tumor-reactive CD8 T cells and may provide an exploratory readout of antitumor immune engagement. However, CD39 expression is biologically heterogeneous and should not be interpreted as a standalone clinical biomarker. In solid tumors, co-expression of CD39 and CD103 identifies tumor-reactive CD8 subsets, suggesting potential translational relevance for perioperative immune monitoring [23].

Stem-like TCF-1^+^ CD8 precursors: TCF-1^+^ CD8 T cells represent a stem-like progenitor population with self-renewal capacity and the ability to generate effector subsets in response to checkpoint blockade. These cells have been associated with durable antitumor immunity and may expand with effective neoadjuvant immune priming in early translational studies, although their perioperative relevance in RCC remains exploratory [24,25].

KIM-1: KIM-1 is a circulating marker of renal tubular injury that has been evaluated for prognostic enrichment in RCC. In IMmotion010, higher baseline levels were associated with increased recurrence risk, suggesting a potential use for risk stratification. However, its predictive value remains unproven, and KIM-1 currently represents an exploratory biomarker requiring prospective validation [26].

Sarcomatoid phenotype: The sarcomatoid phenotype represents a biologically distinct, immune-inflamed subgroup of RCC characterized by high PD-L1 expression and consistent sensitivity to immune checkpoint inhibition. Although these features suggest potential relevance for perioperative treatment intensification, sarcomatoid differentiation has not been validated as a biomarker for therapeutic selection in the perioperative setting [27,28].

Circulating tumor DNA (ctDNA): ctDNA is emerging as a promising marker of MRD and early relapse risk in RCC. Early studies suggest that detectable ctDNA after surgery may identify patients at higher risk of recurrence, although detection rates are limited by the characteristically low tumor DNA shedding observed in RCC, particularly in localized disease. No standardized thresholds for ctDNA positivity or ctDNA-guided perioperative strategies have been established, and ctDNA remains an exploratory biomarker requiring prospective validation [29,30].

These biomarkers are not yet ready for routine clinical decision-making outside of prospective clinical trials because they lack sufficient analytical and clinical validation, standardization of assays, reproducibility across platforms, and robust prospective evidence demonstrating clinical utility.

From Biology to Design: Perioperative “Sandwich” Strategies

Integrating both treatment windows - neoadjuvant priming followed by adjuvant consolidation - may maximize efficacy by enhancing T-cell activation before surgery and suppressing MRD afterwards. However, sufficient pre-operative exposure is crucial: the phase III PROSPER RCC trial, which tested only a single neoadjuvant nivolumab dose before adjuvant therapy, failed to improve RFS, suggesting that inadequate dosing and adherence undermined the biological rationale [31].

In this framework, RCC immunobiology supports perioperative strategies that (1) prime and diversify tumor-reactive T cells with neoadjuvant therapy, (2) maintain immune pressure in the adjuvant setting, and (3) adapt treatment intensity using translational readouts (e.g., CD39^+^/TCF-1^+^ CD8, KIM-1, ctDNA). A plausible next step should be a biomarker-integrated, IO-TKI sandwich design with adequate neoadjuvant exposure to induce immune priming, followed by adjuvant continuation to suppress MRD.

Clinical evidence

Neoadjuvant Trials: Feasible, Informative, and Evolving

Overview and mechanistic rationale: Neoadjuvant therapy in RCC remains investigational, yet accumulating early-phase evidence supports its feasibility, safety, and translational relevance. Both ICIs and tyrosine kinase inhibitors (TKIs), alone or in combination, have been administered prior to nephrectomy without delaying surgery, even in anatomically challenging cases such as IVC thrombus. While radiographic responses have been modest, these studies highlight the potential of neoadjuvant therapy to facilitate surgery and provide unique opportunities for biomarker discovery and immune profiling [3,9].

Evidence from single-agent immunotherapy trials: The first published prospective study of neoadjuvant immunotherapy in RCC was a single-center phase II trial from MSKCC, in which Carlo et al. evaluated nivolumab in 18 patients with locally advanced clear-cell RCC. Patients received nivolumab every two weeks for four cycles; 16/18 completed all four, and all underwent surgery without delay. While no major radiographic responses were observed, correlative analyses demonstrated increased immune infiltration and tumor microenvironment remodeling, highlighting the value of the neoadjuvant setting as a biologic discovery platform [17]. These findings were later corroborated by a separate single-arm phase II trial from Johns Hopkins, where Gorin et al. administered three doses of neoadjuvant nivolumab to 17 patients with high-risk non-metastatic RCC. All patients proceeded to surgery as scheduled, with no unexpected complications or delays, and translational analyses again confirmed enhanced intratumoral immune infiltration despite limited radiographic shrinkage. Two-year metastasis-free survival was 85.1% and OS was 100% [32]. Together, these studies reinforce the safety and feasibility of neoadjuvant PD-1 blockade while emphasizing its primary role as a biologic rather than cytoreductive strategy.

Rationale and evidence for IO-TKI combinations: Combination regimens with IO and TKIs have also shown promise. In the NeoAvAx trial, 40 patients with high-risk localized RCC received avelumab plus axitinib for 12 weeks prior to surgery. The objective response rate (ORR) was 30% (all partial responses), with additional tumor downsizing observed in many patients. Translational analyses demonstrated increased CD8^+^ T-cell infiltration and PD-L1 expression following therapy. Notably, spatial transcriptomics profiling revealed that patients who remained disease-free had distinct CD8^+^CD39⁺ T-cell populations, suggesting that recurrence risk may be linked to the degree of tumor-specific immune engagement [18]. Similarly, the NEOTAX study tested toripalimab plus axitinib in patients with locally advanced RCC and IVC thrombus. Forty-four percent of patients achieved thrombus downstaging, and thrombus length reduction was seen in nearly all cases, facilitating less invasive surgical approaches [19]. A phase II trial evaluating sitravatinib plus nivolumab reported an ORR of 11.8% but demonstrated strong immune activation, including upregulation of interferon-γ signaling pathways. At two years, DFS remained high at 88%, reinforcing the biologic activity of the regimen despite limited tumor shrinkage [11].

TKI-only approaches and evidence in IVC tumor thrombus: TKI-only strategies have been investigated primarily for surgical facilitation. The NAXIVA trial enrolled 20 patients with RCC and IVC thrombus to receive axitinib for up to eight weeks before surgery. Thrombus length reduction was observed in 75% of patients, Mayo level downstaging in 35%, and surgical de-escalation in 41% [10]. In a phase II trial of neoadjuvant cabozantinib in 17 patients with locally advanced, non-metastatic clear-cell RCC, 35% achieved a partial response, median tumor shrinkage was 26%, and one-year DFS was 82%. Paired analyses showed increased intratumoral CD8^+^ and TCF1^+^ “stem-like” CD8^+^ niches, consistent with immune priming [33]. The PADRES study explored axitinib as a strategy to enable nephron-sparing surgery. Among 26 patients with anatomically complex RCC initially deemed poor candidates for partial nephrectomy, neoadjuvant axitinib achieved a reduction in tumor diameter (7.5 vs. 6.2 cm) and RENAL score (11 vs. 10), allowing 74% of patients to undergo successful partial nephrectomy with high negative margin rates [34]. These findings suggest that vascular-targeted therapy may help facilitate technically complex operations, although the supporting evidence derives from small uncontrolled studies, and the impact on long-term oncological outcomes remains uncertain.

Among all settings, patients with IVC tumor thrombus appear to derive the most tangible surgical benefit. Prospective trials and reviews confirm that both TKIs and IO-TKI regimens can reduce thrombus length or level, simplifying resection and occasionally allowing for less invasive surgery. However, no survival advantage has been demonstrated to date [35].

Limitations of current neoadjuvant evidence: Overall, neoadjuvant therapy in localized RCC appears feasible and generally safe. Radiographic responses are modest, and the most consistently observed potential benefit is surgical facilitation in cases with IVC tumor thrombus, although evidence remains limited to small non-randomized phase II studies. Immunotherapy-based approaches provide a useful translational platform for biomarker development, but the evidence base is small, single-arm, and heterogeneous. No randomized neoadjuvant trial has shown an improvement in DFS or OS to date. In line with current guidelines, neoadjuvant therapy should remain investigational and be delivered only within clinical trials [3].

Adjuvant Trials: From TKIs to ICIs

Adjuvant systemic therapy in RCC has undergone a remarkable evolution, transitioning from VEGF- and mTOR-targeted agents to modern ICIs. While VEGFR TKIs and mTOR inhibition dominated the first wave of phase III trials, their disappointing efficacy and toxicity profiles ultimately shifted the focus toward immunotherapy.

Early VEGFR TKI trials with limited efficacy and significant toxicity: Early VEGFR TKI studies were largely negative. The ASSURE trial tested sunitinib, sorafenib, or placebo in 1,943 patients and demonstrated no improvement in DFS, with high rates of dose reductions and discontinuations due to toxicity [36]. Similarly, the PROTECT trial of pazopanib failed to meet its primary DFS endpoint at the amended therapeutic dose [37]. In contrast, the S-TRAC trial reported a modest DFS benefit with adjuvant sunitinib (HR 0.76; p = 0.03), but no OS benefit on extended follow-up and substantial grade ≥3 toxicity [38,39]. The ATLAS trial of axitinib versus placebo was stopped early for futility, confirming no DFS advantage [40]. Collectively, these data underscore marginal efficacy and limited tolerability of VEGF blockade in the adjuvant setting.

mTOR inhibition and antibody-based therapies: mTOR inhibition yielded similar results. The EVEREST trial randomized 1,545 patients to everolimus versus placebo; the primary endpoint was RFS, and the study narrowly missed its prespecified statistical threshold (HR 0.85; p = 0.0246 vs. boundary of 0.022), with no OS benefit and higher grade ≥3 toxicity [41]. Other targeted strategies also failed: the ARISER trial of girentuximab (anti-CAIX antibody) in 864 patients showed no difference in DFS (HR 0.97) or OS (HR 0.99), with only a small exploratory signal in the tumor with very high CAIX expression [42]. These limitations paved the way for ICIs.

ICIs: The KEYNOTE-564 trial was a phase III study enrolling 994 patients with high-risk clear cell RCC (pT2 grade 4 or sarcomatoid features, pT3 or pT4, node-positive disease, or M1 NED). One year of pembrolizumab improved DFS (HR 0.72; 95% CI 0.59-0.87), and longer-term follow-up confirmed a significant OS benefit (HR 0.62; 95% CI 0.44-0.87), with manageable toxicity [6,22].

Other phase III ICIs trials were negative or inconclusive. CheckMate-914 Part A tested one cycle of ipilimumab plus eight cycles of nivolumab in 816 patients but failed to meet its primary DFS endpoint (HR 0.92; p = 0.53), with 32% discontinuation due to toxicity. CheckMate-914 Part B evaluated nivolumab monotherapy versus placebo and was also negative (HR 0.86; 95% CI 0.62-1.20). Exploratory analyses suggested numerical trends toward benefit in patients with ≥pT3 disease, sarcomatoid features, or PD-L1 ≥1%, but these were not statistically significant [8,43]. Notably, PD-L1 positivity was less frequent in CheckMate-914 (~10%) compared to KEYNOTE-564 (~25%), suggesting that immune contexture may partly explain differential efficacy.

The IMmotion010 trial randomized 778 patients to atezolizumab or placebo for one year but failed to improve DFS (HR 0.93; 95% CI 0.75-1.15). Further exploratory analyses showed that high baseline circulating KIM-1 is associated with worse prognosis but improved clinical outcomes with atezolizumab, compared to placebo [7,26]. Finally, the ongoing RAMPART trial is testing durvalumab alone or with tremelimumab versus active surveillance in a multi-arm adaptive platform; its biomarker-integrated design may provide critical insights into optimizing adjuvant immunotherapy [44].

Current adjuvant evidence: Taken together, these trials underscore the complex and evolving role of adjuvant systemic therapy in RCC. While pembrolizumab represents the first practice-changing advance, its benefit appears restricted to carefully defined high-risk populations. Most VEGFR, mTOR, and antibody-based approaches have not improved DFS or OS, reinforcing the need for precision, biomarker-enriched designs. The major randomized adjuvant trials are summarized in Table 1.

**Table 1:** Adjuvant Systemic Therapy Trials in Localized RCC 4/2: four weeks on treatment followed by two weeks off; BICR: blinded independent central review; BID: twice daily; ccRCC: clear-cell renal cell carcinoma; cont: continuous daily dosing; DFS: disease-free survival; FU: follow-up; HR: hazard ratio; N: total number of enrolled patients; NED: no evidence of disease; NR: not reported; OD: once daily; OS: overall survival; RCC: renal cell carcinoma; RFS: recurrence-free survival; UISS: UCLA Integrated Staging System

Clinical Trial	Study Design	Stage for Study Entry	Treatment Schedule	Median FU (Months)	N (Total)	Histology	Primary Endpoint	DFS/RFS	OS
KEYNOTE-564 [6,22]	Phase III, randomized, double-blind (pembro vs placebo)	pT2 (G4/sarcomatoid), pT3, pT4, N+, or M1-NED (metastatic resected to no evidence of disease within 1 year after nephrectomy)	Pembrolizumab 200 mg IV q3w × 17 cycles (~1 year)	57.2	994	ccRCC	DFS (BICR)	HR 0.72 (0.59-0.87); p < 0.001	HR 0.62 (0.44-0.87), p = 0.0024
CheckMate-914 Part A [8]	Phase III, randomized, double-blind (nivo + ipi vs placebo)	pT2 (high grade), pT3, pT4 or N+ M1-NED excluded.	Nivolumab 240 mg IV q2w × 12 (24 weeks) + ipilimumab 1 mg/kg IV q6w × 4 (24 weeks)	37	816	ccRCC	DFS	HR 0.92 (0.71-1.19); p = 0.53	NR
CheckMate-914 Part B [43]	Phase III, randomized, double-blind (nivo vs placebo)	pT2 (high grade), pT3, pT4 or N+ M1-NED excluded.	Nivolumab 240 mg IV q2w × 12 (24 weeks)	27	825	ccRCC	DFS	HR 0.87 (0.62-1.21), p = 0.40	NR
IMmotion010 [7]	Phase III, randomized, double-blind (atezolizumab vs placebo)	pT2 (G4/sarcomatoid), pT3, pT4, N+, or M1-NED (metastatic resected to no evidence of disease within 1 year after nephrectomy)	Atezolizumab 1,200 mg IV q3w × 16 cycles (~1 year)	44.7	778	Predominantly ccRCC (± variants)	DFS	HR 0.93 (0.75-1.15), p = 0.50	NR
ASSURE [36]	Phase III, double-blind, randomized (sunitinib, sorafenib, placebo)	pT1b G3-4, pT2, pT3, pT4, N1	Sunitinib: 50 mg PO OD, 4/2, ×54 weeks; Sorafenib: 400 mg PO BID cont ×54 weeks	71	1943	Mixed (majority ccRCC)	DFS	Sunitinib: HR 1.02 (0.85-1.23), p = 0.80. Sorafenib: HR 0.97 (0.80-1.17), p = 0.72	Sunitinib: HR 1.06 (0.78-1.45); p = 0.66. Sorafenib HR 0.80 (0.58-1.11) p = 0.12
S-TRAC [38,39]	Phase III, double-blind, randomized (sunitinib vs placebo)	pT3 N0/N1, pT4 N0/N1 (UISS high risk)	Sunitinib 50 mg PO OD, 4/2 for one year	64.8	615	ccRCC	DFS (BICR)	HR 0.76 (0.59-0.98), p = 0.03	HR 0.92 (0.71-1.20); p = 0.51
PROTECT [37]	Phase III, double-blind, randomized (pazopanib vs placebo)	pT2 (grade 3-4), pT3/4 any grade, N+	Pazopanib 800 mg PO OD ×1 year (amended to 600 mg)	76	1538	Mixed (majority ccRCC)	DFS	HR 0.86 (0.70-1.06), p = 0.165	HR 1.0 (0.80-1.26), p > 0.9
ATLAS [40]	Phase III, double-blind, randomized (axitinib vs placebo)	≥pT2 and/or N+, any grade	Axitinib 5 mg PO BID cont for up to three years (minimum planned one year)	52	724	ccRCC	DFS	HR 0.87 (0.66-1.15), p = 0.32	NR
SORCE [45]	Phase III, double-blind, randomized (sorafenib vs placebo)	Intermediate/high risk (Leibovich Risk)	Sorafenib 400 mg PO BID cont for three years vs one year	78	1,711	Mixed (majority ccRCC)	DFS	HR 1.01 (0.88-1.17), p = 0.95	HR 0.97 (0.80-1.17), p = 0.76
EVEREST [41]	Phase III, double-blind, randomized (everolimus vs placebo)	Intermediate-high/very high risk	Everolimus 10 mg PO OD cont for 54 weeks	76	1,545	Mixed	RFS	HR 0.85 (0.72-1.00), p = 0.0246 (did not meet boundary)	HR 0.85 (0.64-1.14), p = 0.30
ARISER [42]	Phase III, double-blind, randomized (girentuximab vs placebo)	pT3/4Nx/N0M0, pTanyN+M0, pT1b/2Nx/N0M0 grade ≥3	Girentuximab IV 50 mg week 1, then 20 mg IV weekly weeks 2-24 (24 weeks)	54	864	ccRCC	DFS	HR 0.97 (0.79-1.18), p = 0.73	HR 0.99 (0.74-1.32), p = 0.96

Perioperative and Sandwich Strategies: Blurring the Lines

The boundary between neoadjuvant and adjuvant therapy is being reshaped by perioperative approaches, where systemic treatment begins before surgery and continues afterwards. This strategy aims to unite the immune-priming advantages of neoadjuvant therapy with the micrometastatic control of adjuvant treatment - effectively maintaining systemic pressure across the perioperative window.

The only completed phase III trial of perioperative ICI in RCC is PROSPER RCC. This study randomized 819 patients with high-risk, non-metastatic RCC (≥cT2 or cN+; any histology; selected M1-NED allowed) to perioperative nivolumab (one pre-operative dose followed by nine post-operative cycles) versus nephrectomy with surveillance. The trial was stopped early for futility: RFS was not improved (HR 0.94; 95% CI 0.74-1.21; p = 0.32) [31]. Approximately 53% completed all planned doses. PROSPER provided design lessons for future studies, including the possible need for adequate neoadjuvant exposure, maximizing adherence, and biologically driven endpoints.

Building on these insights, multiple phase II and ongoing studies are exploring more intensive “sandwich” strategies, in which patients receive a defined course of neoadjuvant IO or IO-TKI therapy, undergo surgery, and then resume systemic treatment post-operatively. This model aims to maximize immune engagement while sustaining post-surgical surveillance. A notable example is the combination of lenvatinib plus pembrolizumab, an effective metastatic RCC treatment now under study in the perioperative setting [46]. Such regimens may be particularly promising in biologically aggressive scenarios, such as sarcomatoid differentiation, which shows superior responsiveness to IO combinations in advanced RCC, and venous tumor thrombus, where neoadjuvant IO-TKI has produced thrombus downstaging and facilitated resection [10,19,28].

The rationale for perioperative strategies is to maintain continuous immune engagement across surgery, minimizing the perioperative window of vulnerability for micrometastatic disease. If tolerability is acceptable, sandwich regimens could deliver both rapid tumor shrinkage and durable recurrence prevention, particularly when coupled with emerging biomarkers such as KIM-1, radiomics, and CD8⁺/CD39⁺ immune phenotypes.

Ongoing perioperative trials, including adaptive platform designs, should clarify optimal sequence, duration, and combinations. For now, perioperative therapy is a conceptual evolution that integrates the strengths of neoadjuvant and adjuvant strategies within a biomarker-informed framework. Neoadjuvant and perioperative studies, including ICI, TKI, and IO-TKI regimens, are summarized in Table 2.

**Table 2:** Neoadjuvant and Perioperative Systemic Therapy Trials in Localized RCC ccRCC: clear-cell renal cell carcinoma; DFS: disease-free survival; FU: follow-up; HR: hazard ratio; IVC: inferior vena cava; MFS: metastasis-free survival; mo: months; MPR: major pathological response; N: total number of enrolled patients; NR: not reported; ORR: objective response rate; OS: overall survival; PN: partial nephrectomy; PR: partial response; RCC: renal cell carcinoma; RFS: recurrence-free survival; RN: radical nephrectomy; VTT: venous tumor thrombus

Trial Name	Study Design	Stage at Study Entry	Treatment	Primary Endpoint	N	Median Follow-Up (Months)	Main Finding	RFS/DFS/MFS	OS	Pathologic Response	Surgery Impact
Carlo et al. [17]	Phase II, single-arm, open-label, neoadjuvant	High risk of recurrence RCC (12-year probability of metastases of >= 20%, as per a pre-operative nomogram)	Nivolumab IV q2w ×4 pre-op	Feasibility/safety	18	NR	Safe; no surgical delays; 16/18 completed all four cycles	NR	NR	No pCR; no MPR; increased intratumoral immune infiltration	No delay; feasible nephrectomy
Gorin et al. [32]	Phase II, single-arm, open-label, neoadjuvant	Non-metastatic, high-risk ccRCC (T2a-T4NanyM0 or TanyN1M0)	Nivolumab 3 mg/kg IV q2w ×3 pre-op	Feasibility/safety	17	NR	Safe; increased intratumoral T cell infiltration	MFS: 85.1% at two years (exploratory)	100% at two years (exploratory)	No MPR; limited necrosis	No delay; all proceeded to planned surgery; all had stable disease
NeoAvAx [18]	Phase II, single-arm, open-label, neoadjuvant	High-risk non-metastatic ccRCC; cT1b-T4, cN0-N1, M0, grade 3-4.	Avelumab 10 mg/kg IV q2w + axitinib 5 mg PO BID ×12 weeks pre-op	ORR (RECIST)	40	NR	ORR 30% (PR); increased CD8 and PD-L1; CD39+CD8 signal associated with recurrence risk (exploratory)	NR	NR	No pCR reported; immune activation on paired tissue	No delay; tumor downsizing
Karam et al. [11]	Phase II, single-arm, open-label, neoadjuvant	Locally advanced ccRCC, non-metastatic (cT2-cT3, node positive allowed)	Sitravatinib 120 mg PO OD lead-in ×2 weeks, then nivolumab 240 mg IV q2w ×4-6 weeks pre-op	ORR (RECIST)	17	NR	ORR 11.8%; strong immune activation, including IFN-γ pathway upregulation	DFS 88% at two years (exploratory)	NR	No pCR; immune activation observed	No delay
NAXIVA [10]	Phase II, single-arm, open-label, neoadjuvant	Locally advanced or metastatic ccRCC with venous tumor thrombus (VTT; involving renal vein or IVC), all deemed suitable for nephrectomy and thrombectomy.	Axitinib 5 mg PO BID, uptitrated to 7-10 mg PO BID; ×8 weeks pre-op	Reduction in thrombus extent/level	20	NR	VTT length reduction in 75%; Mayo level downstaging in 35%; less invasive surgery in 41%; PR 14.3% (RECIST)	NR	NR	NR	Facilitated resection and vascular control
NEOTAX [19]	Phase II, single-arm, open-label, neoadjuvant	Non-metastatic ccRCC (T2-T3N0-1M0) or ccRCC with IVC tumor thrombus	Toripalimab 240 mg IV q3w + axitinib 5 mg PO BID ×12 weeks pre-op	Downstaging rate of IVC-TT	25	23.3	VTT downstaging 44%; median thrombus length −2.3 cm; surgical plan modified in 61.9%; PFS 25.3 months; one-year 89.1%, 2-year 54.8% (exploratory)	NR	NR	NR	No surgical delay; enabled a less morbid approach
PADRES [34]	Phase II, single-arm, open-label, neoadjuvant	cT1–T2 complex renal masses (nephron-sparing intent)	Axitinib 5 mg PO BID ×8 weeks pre-op	Feasibility of partial nephrectomy	27	8.5	PN completed in 74.1%; PR 33% (RECIST)	NR	NR	NR	Enabled PN in anatomically complex tumors
Bilen et al. [33]	Phase II, single-arm, open-label, neoadjuvant	≥cT3Nx or TanyN+ M0, or initially deemed unresectable	Cabozantinib 60 mg PO OD ×12 weeks pre-op	ORR (RECIST)	17	25	ORR 35%; immune remodeling with increased intratumoral CD8+ and TCF-1+ CD8+ niches	DFS 82.4% at one year	94.1% at 1 year	No pCR; immune remodeling	Increased feasibility of nephrectomy/PN
Cowey et al. [47]	Phase II, single-arm, open-label, neoadjuvant	≥cT2 RCC, including localized and metastatic (limited)	Sorafenib 400 mg PO BID ×5 weeks pre-op (median 33 days)	Safety, feasibility, and tumor burden	30	NR	Median tumor shrinkage9.6%; 29/30 completed therapy; 25 proceeded to resection; well tolerated	NR	NR	No pCR; no reported MPR; modest tumor shrinkage in most	No excess perioperative morbidity
Hellenthal et al. [48]	Phase II, single-arm, open-label, neoadjuvant	cT1-T3, N0/N1, M0, any histology; some included M1 disease	Sunitinib 37.5 mg PO OD ×12 weeks pre-op	Safety	20	NR	Sunitinib is safe pre-operatively; 85% had tumor shrinkage; median -11.8% tumor diameter reduction	NR	NR	No pCR; PR in 1/20; others had stable disease	No significant increase in perioperative complications
Karam et al. [49]	Phase II, single-arm, open-label, neoadjuvant	cT2-T3, N0, M0, ccRCC	Axitinib 5 mg PO BID ×12 weeks pre-op	ORR (RECIST)	24	NR	RECIST PR 46%; Median tumor size reduction of 28%; no on-treatment progression	NR	NR	No pCR	No surgical delay; PN or RN performed per surgeon's discretion
Rini et al. [50]	Phase II, single-arm, open-label, neoadjuvant	Localized ccRCC patients scheduled for radical or partial nephrectomy, including complex masses at high risk for partial nephrectomy failure.	Pazopanib 800 mg PO OD ×8-16 weeks pre-op	Rate of successful PN after therapy	25	NR	Tumor complexity reduced (RENAL score reduction in 71%); volume reduction in 92%; 6/13 converted to PN	NR	NR	NR	Improved feasibility of nephron-sparing surgery
Ornstein et al. [51]	Phase Ib, single-arm, open-label, perioperative (neoadjuvant + adjuvant)	cT2b-4 and/or N1, M0	Durvalumab 10mg/kg IV q2w ± Tremelimumab 1mg/kg IV q6w: 1 pre-op dose, then adjuvant durvalumab ± tremelimumab	Safety/feasibility	29	NR	Perioperative durvalumab was feasible; tremelimubar increased toxicity	NR	NR	NR	No treatment-related surgical delays
PROSPER RCC [31]	Phase III, randomized, open-label, perioperative vs observation.	High-risk, non-metastatic RCC (≥cT2 or cN+; any histology; selected M1-NED allowed)	Nivolumab 480 mg IV: 1 pre-op dose + 9 q4w post-op	Investigator-assessed RFS	819	30.4	No RFS benefit; treatment completion 53%	RFS: HR 0.94 (0.74-1.21); p = 0.32	OS not improved (immature)	NR	No significant surgical delays

Future directions

The future of perioperative systemic therapy in RCC will hinge on three priorities: precision in patient selection, optimization of treatment sequencing and intensity, and integration of biomarker discovery into adaptive trial designs.

Biomarker-Guided Patient Selection

Adjuvant pembrolizumab, validated by the KEYNOTE-564 trial, is the first systemic therapy to demonstrate both disease-free and OS benefit after nephrectomy, establishing a new standard for high-risk clear cell RCC. Yet nearly half of placebo-treated patients remain recurrence-free at four years, while more than one-third of pembrolizumab-treated patients relapse [22]. This dual reality underscores the challenge of overtreatment in some patients cured by surgery alone and undertreatment in others with biomarker-negative disease, highlighting the need for precision selection tools. Circulating biomarkers such as kidney injury molecule-1 (KIM-1), MRD assays, immune gene signatures, and radiomics/AI models are emerging as promising candidates for guiding adjuvant decisions [52-54].

Optimizing Timing and Sequencing

Timing and structure of perioperative therapy remain unsettled. The phase III PROSPER trial, which evaluated perioperative nivolumab with a single-dose neoadjuvant component followed by adjuvant therapy, did not improve RFS [31]. This outcome underscores persistent uncertainty about how much neoadjuvant exposure - or which perioperative sequence - is required to meaningfully influence long-term disease control. Conversely, the adjuvant-only model risks missing a biologically favorable pre-operative window. The field is therefore shifting toward “sandwich” strategies, in which intensive neoadjuvant IO or IO-TKI therapy primes immunity, uninterrupted continuation through surgery mitigates perioperative immunosuppression, and extended adjuvant therapy sustains micrometastatic control. These designs may be particularly relevant in aggressive subsets such as sarcomatoid RCC or in IVC tumor thrombus, where biology is aggressive and neoadjuvant IO-TKI can facilitate resection [52].

Adaptive and Platform Trial Designs

A major innovation is the shift in trial methodology. Rather than relying on static, binary phase III designs that risk futility, next-generation studies are increasingly adopting adaptive, biomarker-integrated platform designs capable of evaluating multiple strategies.

The RAMPART trial (NCT03288532) exemplifies this approach, testing durvalumab alone or with tremelimumab against surveillance in a multi-arm, multi-stage design with adaptive expansion or closure based on interim analyses [44]. Embedding translational endpoints within such platforms accelerates identification of active regimens, supports real-time biomarker validation, and aligns perioperative therapy development with the biological heterogeneity of RCC.

Finally, response-directed therapy is an emerging paradigm in RCC, echoing progress in melanoma (OpACIN-neo, NADINA) and urothelial cancer (CheckMate-274). Standardizing pathological response criteria after neoadjuvant therapy could enable escalation or de-escalation of systemic treatment based on residual viable tumor, turning nephrectomy specimens into a real-time decision tool and providing validated surrogate endpoints for future trials [52].

Summary

The next decade of perioperative RCC therapy will be defined less by single “positive” or “negative” trials and more by an integrated paradigm: validated biomarkers, adaptive trial designs, sandwich sequencing of IO and IO-TKI regimens, and response-guided tailoring. Pembrolizumab has opened the door, but the future lies in ensuring that only the right patients walk through it, for the right duration, with the right combination, guided by robust biomarkers and adaptive evidence.

## Conclusions

The perioperative management of localized RCC is entering a transformative phase. Adjuvant pembrolizumab has established a new standard of care for high-risk clear cell RCC, supported by both DFS and OS benefit. Yet the heterogeneity of results with other checkpoint inhibitors, the negative outcome of PROSPER, and the risk of overtreating patients cured by surgery alone demand greater precision.

Neoadjuvant and perioperative approaches remain investigational yet biologically compelling - offering immune priming, potential surgical facilitation in anatomically complex cases, and a window for mechanistic discovery. The field should now move beyond one-size-fits-all care: integrate clinicopathological risk with molecular and circulating biomarkers, embed these within adaptive platforms, and tailor the sequence, intensity, and duration of therapy. Such personalization should maximize oncological benefit while minimizing toxicity and preserving quality of life.
